# The clinical, myopathological, and molecular characteristics of 26 Chinese patients with dysferlinopathy: a high proportion of misdiagnosis and novel variants

**DOI:** 10.1186/s12883-022-02905-w

**Published:** 2022-11-01

**Authors:** Ning Wang, Xu Han, Shengpu Hao, Jingzhe Han, Xiaomeng Zhou, Shuyan Sun, Jin Tang, Yanpeng Lu, Hongran Wu, Shaojuan Ma, Xueqin Song, Guang Ji

**Affiliations:** 1 Department of Neurology, The Second Hospital of Hebei Medical University, 050000 Shijiazhuang, Hebei People’s Republic of China; 2 The Key Laboratory of Neurology (Hebei Medical University), Ministry of Education, 050000 Shijiazhuang, Hebei People’s Republic of China; 3 MyGenosticsInc, Beijing, China

**Keywords:** Dysferlin, LGMD R2, Atypical asymptomatic hyperCKemia, Muscle pathology

## Abstract

**Background:**

Dysferlinopathy is an autosomal recessive muscular dystrophy caused by pathogenic variants in the dysferlin (*DYSF*) gene. This disease shows heterogeneous clinical phenotypes and genetic characteristics.

**Methods:**

We reviewed the clinical and pathological data as well as the molecular characteristics of 26 Chinese patients with dysferlinopathy screened by immunohistochemistry staining and pathogenic variants in *DYSF* genes.

**Results:**

Among 26 patients with dysferlinopathy, 18 patients (69.2%) presented as Limb-girdle Muscular Dystrophy Type R2 (LGMD R2), 4 (15.4%) had a phenotype of Miyoshi myopathy (MM), and 4 (15.4%) presented as asymptomatic hyperCKemia. Fifteen patients (57.7%) were originally misdiagnosed as inflammatory myopathy or other diseases. Fifteen novel variants were identified among the 40 variant sites identified in this cohort.

**Conclusion:**

Dysferlinopathy is a clinically and genetically heterogeneous group of disorders with various phenotypes, a high proportion of novel variants, and a high rate of misdiagnosis before immunohistochemistry staining and genetic analysis.

**Supplementary Information:**

The online version contains supplementary material available at 10.1186/s12883-022-02905-w.

## Introduction

Dysferlinopathy is an autosomal recessive muscular dystrophy caused by pathogenic variants in the *DYSF* gene, which is located on chromosome 2p13 and spans a genomic region of over 230 kbp consisting of 55 exons [1, 2]. It encodes the dysferlin, a transmembrane protein involved in membrane repair [3], Ca^2 + ^ signaling pathway [4], cell adhesion [5], and T-tubule formation [6]. Pathogenic variants in *DYSF* lead to abnormal muscle wasting and cause different clinical phenotypes mainly including limb-girdle muscular dystrophy type R2 (LGMD R2) and Miyoshi myopathy (MM) [7]. Both LGMD R2 and MM develop in young adults with a slow course and elevated levels of creatine kinase (CK). However, weakness and atrophy of the muscle involved were different in LGMD R2 and MM, with the former mainly affecting the pelvic and shoulder girdle muscles, while the latter mainly affecting the posterior compartment of the leg [8]. LGMD R2 is the second most common form of LGMD in Western countries [9] and Japan [10] and is the most prevalent genotype of LGMD in China [11].

This disease exhibits a variety of dystrophic features in muscle pathology, including fibrosis, necrosis and changes in fiber size, and sometimes shows inflammatory infiltrates [12], which are easily misdiagnosed as inflammatory myopathy because of multiple overlapping clinical features [13]. Corticosteroid treatment in these patients may lead to irreversible muscle damage, and it is difficult to distinguish between dysferlinopathy and inflammatory myopathy when the diagnosis is based solely on routine clinicopathological examination [14]. Immunohistochemistry (IHC) showed highly reduced expression of dysferlin protein, which is still a mandatory criterion for a positive diagnosis [15]. But since similar manifestations can also be seen in some other secondary myopathies, gene diagnosis remains the gold standard [16]. In this study, we reviewed the clinical and molecular characteristics of 26 Chinese patients with dysferlinopathy screened by immunohistochemistry and genetic analysis, and identified a high proportion of novel variants which expand the genetic spectrum of dysferlinopathy. Recently Zhong et al. reported 245 dysferlinopathy patients in 2021, although, our data further supplemented their study [11] and emphasized the importance of differentiation from inflammatory myopathy.

## Methods

### Patient selection criteria and clinical evaluation

We retrospectively reassessed clinical data of 26 Chinese patients (including two patients previously reported) [17, 18] from unrelated families with muscle biopsy in our hospital followed up based on the following inclusion criteria: (1) loss or strong reduction of dysferlin expression evidenced by immunohistochemistry on muscle biopsy and (2) variants identified in the *DYSF* gene (n = 26). According to the reference range of different hospitals, the CK levels were normalized as x-fold of the upper limit of normal values.

Standard histological methods were used to examine muscle slices. Muscle biopsy specimens were taken from the patients’ biceps brachii, quadriceps femoris, or gastrocnemius muscles after informed consent. Biopsied skeletal muscles were flash-frozen in isopentane chilled by liquid nitrogen. The histopathological analysis includes hematoxylin-eosin (HE), modified Gomori trichrome (MGT), succinate dehydrogenase (SDH), myosin ATPase, acid phosphatase (ACP), NADH-tetrazolium reductase (NADH-TR), oil red O (ORO), and periodic acid-Schiff (PAS). Morphological determination of muscle specimens was finished under light microscopy. Anti-Dysferlin antibody (Abcam, JAI-1-49-3, rabbit, United Kingdom; dilution (1:100) was used to perform immunohistochemistry on muscle biopsies.

### Genomic analysis

The screening of *DYSF* variants was conducted as described previously [11, 19] (transcript number NM_003494.4). Most of the clinical exome sequencing analysis were accomplished by MyGenosticsInc, Beijing, China.

### ACMG/AMP rules used to classify the variants

The variants identified in patients are classified according to ACMG/AMP guidelines, which are currently the standard in modern genetics. ACMG/AMP codes that were used for the classification have been provided for each variant in a Supplementary Table [see Additional file 1]. In the Chinese dysferlinopathy cohort, several criteria of the ACMG/AMP guidelines were modified as follows: PVS1: nonfunctional variants occur in critical genes, including nonsense, frameshift, splice, deletion/repeat and start codon variants; PS1: A variant with the same amino acid change but a different nucleotide change as a known pathogenic variant; PS3: In vivo and in vitro functional assays have established that variants cause impaired gene function; PM2: Rare or missing variants in the population database; Referring to the SVI Recommendation for in trans Criterion PM3 (Version 1.0), the PM3 score was given; The standards for PP3 recommended by the 2019 Association for Clinical Genomic Science ACGS are: REVEL ≥ 0.7, or > 2/3 of tools predicted to be harmful; For BP4, the criteria are: REVEL ≤ 0.4, or > 2/3 of the tools are predicted to be harmless and the variant position is not conservative, or no tool is predicted to be harmless; PP4: The disease associated with the variant was highly consistent with the patient’s symptoms and family history. All patients underwent immunohistochemical analysis to observe protein expression. If dysferlin was absent from muscle tissues found by immunohistochemistry in at least two patients with a pathogenic variant, PP4_strong was given (PP4_moderate if absent in one patient, PP4_supporting if decreased in one patient). PM2, BS1, and BA1 allele frequency thresholds were set at 0.02%, 0.5%, and 5%, respectively.

### Statistical analysis

All values were calculated using IBM SPSS Statistics 26.0. Values are presented as the mean ± standard error (SE) unless otherwise stated. ANOVA(ANalysis Of VAriance)was used to test the significance of differences in age of onset and serum creatine kinase (CK) level between the different types of dysferlinopathy. A value of *P* ≤ 0.05 was considered statistically significant (two-tailed).

## Results

### Clinical Data

The 26 patients came from nine provinces in China. Among the 26 patients, 12 were females and 14 were males (Table 1). All patients had normal motor milestones. The average age of onset was 24.5 ± 6.5 years (range 16–37 years). Eighteen patients (69.2%) presented as LGMD R2, 4 (15.4%) as MM, and 4 (15.4%) as asymptomatic hyperCKemia. The corresponding average ages of onset for each of the three types were listed as 25.6 ± 6.8 (range 16–21 years), 21.8 ± 5.1 (range 17–29 years), and 22.0 ± 6.1 (range 16–29 years) (p = 0.41).

Fifteen patients (57.7%) were misdiagnosed as inflammatory myopathy before muscle biopsy (including twelve as LGMD R2, two as MM, and one as asymptomatic hyperCKemia), and 13 of them (86.7%) received corticosteroids, and some also received immunosuppressive drugs. Misdiagnosis of inflammatory myopathy was more frequent in the LGMD R2 group (12 of 18 patients [66.7%]) vs. the MM group (2 of 4 patients [50%]) and the hyperCKemia group (1 of 4 patients [25%]). Three patients were misdiagnosed as viral myocarditis (3 of 4 patients [75%]), all of whom presented as asymptomatic hyperCKemia. The other misdiagnosis includes peripheral neuropathy (n = 1), hepatopathy (n = 1), and arthritis (n = 1).

### Serum CK level

**Fig. 1:**
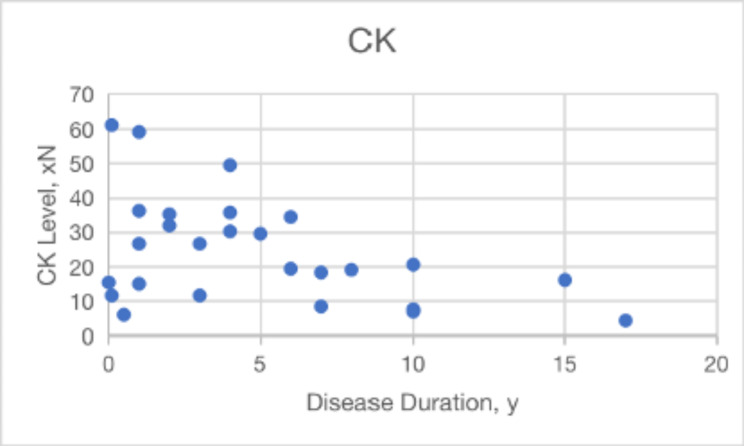
Levels of creatine kinase (CK) during the disease. There is a downward trend in levels over time. Each point indicates one patient’s most recent CK level (N = 26)

The mean ± SD minimal level of CK was 24.8 ± 15.9 xN (range 4-62xN). CK levels tended to drop as the disease progressed (Fig. 1). We discovered no correlation between CK levels and disease development in the three primary phenotypes LGMD R2, MM, and asymptomatic hyperCKemia, and the corresponding CK values of the three types are 22.8 ± 12.1 xN (range 4.3–41.6 xN ), 33.3 ± 20.9 xN (range 8.4–59.3 xN), 23.6 ± 25.4 xN (range 6.0–61.3 xN ) (p = 0.49), respectively.

### Histological and immunohistochemical staining

**Fig. 2:**
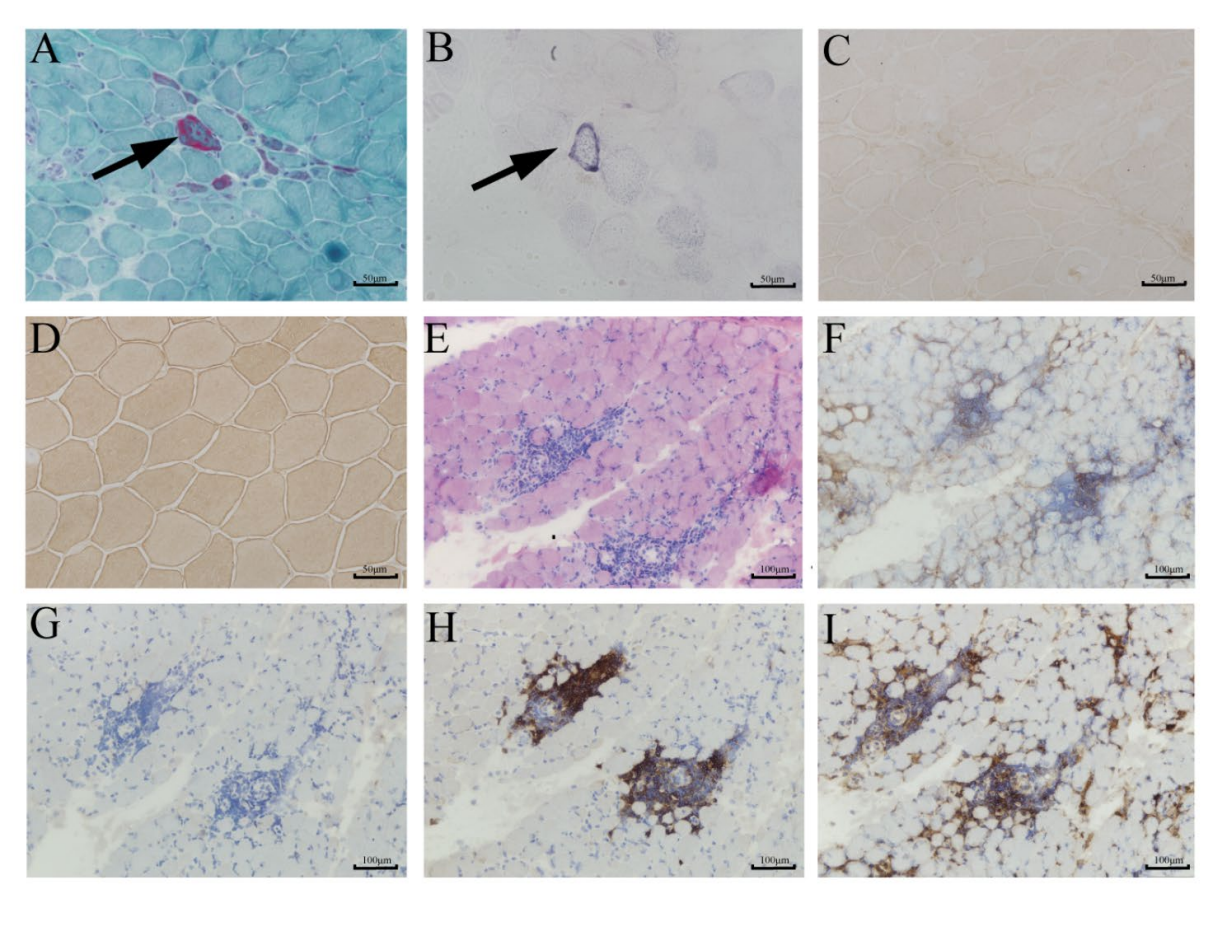
(**A-C**) Muscle biopsy from left quadriceps of Patient 1. (**A**) Modified Gomori trichrome (400×) shows several ragged red fibers (RRF) (arrow). (**B**) Succinate Dehydrogenase (400×) highlights the ragged blue fiber (RBF) (arrow). (**C**) Immunohistochemistry staining of muscle fibers shows a complete absence of dysferlin expression (400×). (**D**) normal control of dysferlin (400×). (**E-I**) Muscle biopsy from left biceps of Patient 13. (**E**) Hematoxylin and eosin (HE) (200×) staining shows that inflammatory cells are seen between muscle fibers. Immunohistochemistry staining of muscle fibers shows (**F**) CD4 positive expression (×200), (**G**) CD8 negative expression (×200), (**H**) CD20 positive expression (×200) and (**I**) CD68 positive expression (×200)

A total of muscle samples corresponding to the 26 patients included were available. The mean ± SD age at biopsy was 29.3 ± 9.0 years (range 16–52 years), and the mean ± SD disease duration was 4.9 ± 4.6 years (range 0–17 years). Samples were retrieved from biceps brachia (n = 11), quadriceps (n = 11), and gastrocnemius (n = 4) muscles. Muscle biopsies from most patients showed markedly increased variation in fiber diameter, necrotic and regenerating fibers, splitting fibers, fibrosis, and adipose deposition to a variable degree. Ragged red fibers (RRF) and ragged blue fibers (RBF) were observed in 2 patients (Fig. 2 A, B), and vacuolation was observed in 1 patient. Immunohistochemical analysis of most of the patients showed a complete absence of dysferlin expression in patients (Fig. 2 C) compared with the normal control group (Fig. 2D) and a strong reduction of dysferlin expression in 6 patients. Besides, Inflammatory cell infiltration occurred in 5 patients which makes it easily confused with inflammatory myopathy (Fig. 2E-I).

### Variant analysis

Among 26 patients, 25 had 2 variants, either 1 homozygous (n = 4) or 2 compound heterozygous (n = 21) variants identified in the *DYSF* gene, and one had only 1 heterozygous *DYSF* gene variant. Forty different variants were identified (including 15 not previously reported) [Table 1], including 17 missense (5 novel), 10 nonsense (4 novel), 7 exonicframeshifting variants (insertions or deletions, 4 novel), 4 splice variants (0 novel), and 2 exon duplication variants (2 novel). The c.3112 C > T (p. Arg1038Ter) was identified in more than one patient, and the patients who had the same variant came from different provinces.

**Table 1:** Clinical data and pathogenic variants obtained in the cohort

Patient No	Sex	Age at onset	disease duration	CK fold	Pheno-type	Misdiagnose	Dysferlin on IHC	Zygos-ity	Mutation type	Genomic position	Nucleotide changes	Protein change	ACMG/AMP codes	ACMG classification
1	M	26	2	35.3	LGMDR2	polymyositis	-	Het	Canonical-splice	chr2:71708069	c.144 + 1G > A	splicing	PVS1 + PM2 + PP4_ moderate	Pathogenic
								Het	splice	chr2:71753481	c.1273 + 5G > C	splicing	PVS1 + PM2 + PP4_ moderate	Pathogenic
2	M	18	0.1	11.6	HyperCK	viral myocarditis	-	Hom	missense	chr2:71747946*1	c.965T > C	p. L322P	PS1 + PM2 + PM3 + PP4_ moderate	Pathogenic
3	M	16	0.5	6	HyperCK	viral myocarditis	strongly reduced	Het	missense	chr2:71797459	c.3026 A > G*	p. E1009G	PM2 + PP4	VUS
								Het	nonsense	chr:71,797,809	c.3112 C > T	p. R1038X	PVS1 + PS1 + PM2 + PP4_moderate	Pathogenic
4	M	25	0.1	61.3	HyperCK	dermatomyositis	-	Het	nonsense	chr2:71797809	c.3112 C > T	p. R1038X	PVS1 + PS1 + PM2 + PP4_ moderate	Pathogenic
							-	Het	nonsense	chr2:71838459	c.3988 C > T	p. Q1330X	PVS1 + PS1 + PM2 + PP4_ moderate	Pathogenic
5	M	29	0	15.4	HyperCK	viral myocarditis	-	Het	missense	chr2:71801439	c.3286 C > A*	p. R1096S	PM2 + PP4	VUS
							-	Het	nonsense	chr2:71906245	c.5826 C > A*	p. C1942X	PVS1 + PM2 + PM3 + PP4_ moderate	Pathogenic
6	F	24	7	18.3	LGMDR2		-	Het	canonical-splice	chr2:71747339	c.937 + 1G > A	splicing	PVS1 + PS1 + PM2 + PP4_ moderate	Pathogenic
							-	Het	missense	chr2:71797407	c.2974T > C	p. Trp992Arg	PS1 + PM2 + PP4_ moderate	likely Pathogenic
7	M	25	10	6.9	LGMDR2	polymyositis	strongly reduced	Hom	missense	chr2:71753461	c.1165G > A	p. Glu389Lys	PM2 + PM3_Supporting + PP4	VUS
8	M	16	6	19.4	LGMDR2		-	Het	nonsense	chr2:71886125	c.4756 C > T	p. R1586X	PVS1 + PS1 + PM2 + PM3 + PP4_ supporting	Pathogenic
							-	Het	frameshift	chr2:7189462071894620;	c.5316dupC*	p. S1772fs	PVS1 + PM2 + PM3 + PP4_ moderate	Pathogenic
9	F	16	10	20.6	LGMDR2		-	Het	nonsense	chr2:71740998	c.610 C > T	p. Arg204Term	PVS1 + PS1 + PM2 + PP4_ moderate	Pathogenic
							-	Het	duplication	23–30 Exon	exon22-29 suspected duplication variant*		PM2 + PP4	VUS
10	M	27	4	30.3	LGMDR2		-	Het	missense	chr2-71791250	c.2418 C > A*	p. Y806X	PVS1 + PM2 + PM3_strong + PP4_ moderate	Pathogenic
							-	Het	missense	chr2:71896337	c.5525G > A	p. G1842D	PS1 + PM2 + PM3_strong + PP4_ moderate	Pathogenic
11	F	21	1	26.7	LGMDR2	polymyositis	strongly reduced	Hom	missense	chr2:71894601	c.5296G > A	p. Glu1766Lys	PS1 + PM2 + PM3_supporting + PP4_supporting	Pathogenic
12	M	18	4	49.6	LGMDR2	polymyositis	-	Het	canonical-splice	chr2:71747339	c.937 + 1G > A	splicing	PVS1 + PM2 + PP4_ moderate	Pathogenic
								Het	missense	chr2:71896321	c.5509G > A	p. D1837N	PS1 + PM2 + PP4_ moderate	likely Pathogenic
13	F	18	1	36.3	LGMDR2	polymyositis	strongly reduced	Het	missense	chr2:71827854	c.3725G > A	p. R1242H	PS1 + PP4_ supporting	VUS
								Het	missense	chr2:71886111	c.4742G > A	p. R1581H	PS1 + PM2 + PP4_ supporting	likely Pathogenic
14	F	33	2	32	LGMDR2	polymyositis	-	Het	missense	chr2:71892431	c5197A > G	p. I1733V	PS1 + PM2 + PP4_ moderate	likely Pathogenic
								Het	missense	chr2:71894563	c.5258 A > G*	p.H1753R	PM2 + PP4	VUS
15	F	35	1	15	LGMDR2	polymyositis	-	Het	nonsense	Chr2:71742762	c.673 C > T*	p. Q225X	PVS1 + PM2 + PP4_ moderate	Pathogenic
								Het	missense	Chr2:71906214	c.5795T > A*	p.M1932K	PM2 + PP4	VUS
16	F	27	3	26.7	LGMDR2		strongly reduced	Het	nonsense	chr2:71709020	c.156G > A*	p. W52X	PVS1 + PS2 + PM3 + PP4_ moderate	Pathogenic
								Het	missense	chr2:71896779	c.5570 A > G	p.H1857R	PS1 + PM2 + PM3 + PP4_moderate	Pathogenic
17	M	35	8	19.1	LGMDR2	polymyositis	-	Het	frameshift	chr2:71762413	c.1375dupA	p. Met459Asnfs*15	PVS1 + PM2 + PP4_ moderate	Pathogenic
								Het	frameshift	chr2:71825821	c.3648delA*	splicing	PVS1 + PM2 + PP4_ moderate	Pathogenic
18	F	31	17	4.3	LGMDR2	polymyositis	-	Het	missense	chr2:71742844	c.755 C > T	p. T252M	PS1 + PM2 + PP4_ moderate	Likely pathogenic
								Het	duplication		Exon41-52suspected duplication variant*		PM4 + PP4	VUS
19	F	26	10	7.6	LGMDR2		-	Het	frameshift	chr2:71743324–71,743,328	c.808_811del*	p. F271Tfs*16	PVS1 + PM2 + PP4_ moderate	Pathogenic
								Het	nonsense	chr2:71797809	c.3112 C > T	p. R1038X	PVS1 + PS1 + PM2 + PP4_ moderate	Pathogenic
20	M	37	15	16.1	LGMDR2	polymyositis	-	Het	frameshift	chr2:71801368–71,801,370	c.3216_3217delCT	p. L1074Ffs*39	PVS1 + PM2 + PP4_ moderate	Pathogenic
								Het	missense	chr2:71891543	c.5032T > C	p.C1678R	PM2 + PP4	Likely pathogenic
21	M	18	6	34.5	LGMDR2	polymyositis	-	Hom	frameshift	chr2:71891489–71,891,509	c.4979_4998delGTGAGACGGTCGTCGACCTGinsA*	p. G1660Efs	PVS1 + PM2 + PM3_supporting + PP4_moder-ate	Pathogenic
22	M	29	4	35.8	MM	polymyositis	-	Het	canonical-splice	chr2:71795213	c.2643 + 1G > A	splicing	PVS1 + PS1 + PM2 + PP4_ moderate	Pathogenic
								Het	missense	chr2:71816726	c.3352G > A*	p. G1118S	PM2 + PP4	VUS
23	F	21	5	29.6	MM	polymyositis	strongly reduced	Het	missense	chr2:71797407	c.2974T > C	p. W992R	PS1 + PM2 + PP4_ supporting	Pathogenic
								Het	frameshift	chr2:71896814–71,896,814	c.5606dupG	p. R1870Efs*12	PVS1 + PS1 + PM2 + PP4_ supporting	Pathogenic
24	M	17	1	59.3	MM		-	Het	nonsense	chr2:71740998	c.610 C > T	p. Arg204Term	PVS1 + PS1 + PM2 + PM3 + PP4_ moderate	Pathogenic
								Het	nonsense	chr2:71839831	c.4228 C > T	p. Q1410X	PVS1 + PM2 + PM3 + PP4_ moderate	Pathogenic
25	F	20	7	8.4	MM		-	Het	nonsense	chr2:71797809	c.3112 C > T	p. R1038X	PVS1 + PM3 + PM2 + PP4_ moderate	Pathogenic
								Het	nonsense	chr2:71766369	c.1480G > T	p. E494X	PVS1 + PM3 + PP4_moderate + BP4	Pathogenic
26	F	28	3	11.6	LGMDR2	polymyositis	-	Het	canonical-splice	chr2:71747339	c.937 + 1G > A	splicing	PVS1 + PS1 + PM2 + PP4_ moderate	Pathogenic

*, novel variant; -, completely reduction; Het, heterozygous; Hom, homozygous; LGMD R2, limb-girdle muscular dystrophy type R2; MM, Miyoshi Myopathy; HyperCK, asymptomatic hyperCKemia; VUS, the clinical significance is unclear; transcript number (NM_), NM_003494.4

## Discussion

Dysferlinopathy is muscular dystrophy caused by the deficiency of dysferlin protein coding by the *DYSF* gene. Pathologic variants in *DYSF* lead to different clinical phenotypes, mainly including LGMD R2 and MM [20]. LGMD R2 mainly affects the proximal lower extremity muscle tissue in the youth; as the disease progresses, the scapular girdle and upper extremity muscles may also be affected, but the symptoms are mild; the neck and hand muscles are generally spared [21]. This disease is the second most common LGMD in Europe and Japan but is underdiagnosed in China previously [22]. MM is an adult-onset disorder characterized by early-onset gastrocnemius weakness, which is also accompanied by an increase in serum CK concentration [23]. But the onset of MM was found to be earlier than that of LGMD R2 in the Italian population [24]. Other phenotypes associated with dysferlin deficiency have also been identified, including distal anterior myopathy (DACM) (also known as distal tibial onset distal myopathy), and proximal-distal phenotype (PD) (this phenotype may be a proximally rapidly progressive MM) [25, 26] and asymptomatic hyperCKemia. We didn’t observe DACM in our cohort with the highest proportion of LGMD R2, which was consistent with the domestic sample [22] and foreign studies [25].

LGMD R2 is easily misdiagnosed as inflammatory myopathies, especially polymyositis (PM), which is very similar to LGMD R2 in clinical manifestation and muscle pathology. Both LGMD R2 and PM exhibit proximal muscle weakness and significantly elevated muscle enzymes and may show infiltration of immune cells in muscle pathology, but the treatment is different between them [21]. PM is an immune disease that responds well to hormone therapy [27], but glucocorticoids have been reported to exacerbate muscle weakness in LGMD R2 patients, and the damage to the muscle is irreversible [28]. We recommend that it is important to rule out dysferlinopathy before starting corticosteroid courses. Studies have shown that injection of glucocorticoids into the patient’s muscle cell membrane can damage the membrane stability [29], which may lead to an increase in the CK value, and this instability also exacerbates the lack of fibrillin repair capacity [29]. For LGMD R2, however, the focus is on early symptomatic treatment and appropriate exercise, which can slow disease progression and improve motor function. Therefore, the early diagnosis of LGMD R2 is closely related to the prognosis of patients. In our cohort, 5 patients showed inflammatory cell infiltration in muscle pathology. Twelve LGMD R2 patients (66.7%) in this group were misdiagnosed as polymyositis before biopsy, and 10 of them had received corticosteroid therapy, which may affect the level of CK. The CK level of patient 7 still repeatedly increased after corticosteroid therapy; in addition to corticosteroids, Patient 1 also took traditional Chinese medicine, but the CK level stayed at a high level (35.3 times the normal scope). Besides, 2 MM patients (50%) were misdiagnosed as polymyositis and had previously received corticosteroid therapy before the biopsy. The two diseases can be differentiated by analyzing the expression of dysferlin in muscle tissues by IHC. For patients with LGMD R2, IHC analysis showed a lack of dysferlin in the involved muscle fibers, and MHC-I results were negative or low [30].

Besides inflammatory myopathies, dysferlinopathy patients with a history of exercise intolerance or asymptomatic hyperCKemia [25, 31] may be misdiagnosed as metabolic myopathy. CK levels fluctuated in patients with metabolic myopathy but usually stayed at a high level in patients with dysferlinopathy, except in the late stage of the disease because of muscle wasting. In primary hospitals in China, patients with asymptomatic hyperCKemia at first tended to visit the department of cardiology or general medicine. Three patients of our cohort with asymptomatic hyperCKemia (75%) were misdiagnosed as viral myocarditis. Patients with viral myocarditis usually presented with chest pain, shortness of breath, fever, fainting, and palpitations. CK-MB is one of the diagnostic indicators of viral myocarditis. It has been reported that the CK-MB levels of children with viral myocarditis in the acute phase are about 3 times that of the normal control group [32] and CK levels can slightly increase, while in dysferlinopathy the CK but not the CK-MB levels usually increased significantly. Muscle damage in dysferlinopathy also results in an elevated level of liver enzymes (for example, ALT and AST) which may be confused with liver disease. Our study found that patients with asymptomatic hyperCKemia were easily misdiagnosed as myocarditis (75%) and liver disease (25%), indicating insufficient recognition of this disease in primary hospitals in China, especially for doctors of internal medicine.

In addition to the typical dystrophic features, 2 patients in this dysferlinopathy group presented with several ragged red fibers (RRF) seen on histopathological MGT staining. Previous reports have also documented mitochondrial abnormalities in some patients with dysferlinopathy, in which there is an accumulation of subsarcolemmal mitochondria in muscle fibers, including one patient with RRF and paracrystalline mitochondrial inclusions [33, 34]. The mechanisms for the formation of mitochondria abnormalities observed in muscle pathology are undefined. Previous research showed that dysferlin has a ferlin Ca^2+^ domain with a variable affinity for Ca^2+^ and helps regulate the cytoplasmic Ca^2+^ [33], which becomes abnormally high in the absence of dysferlin. Doug M. Turnbull [35] et al. suggested that dysferlin gene variants increased the concentration of cytoplasmic Ca^2+^, leading to mitochondrial aberrations. However, not only do mitochondria regulate cytoplasmic Ca^2+^ levels, but the abnormal elevation of Ca^2+^ would also affect mitochondria, and calcium influx into the cytoplasm would lead to fragmentation of the mitochondrial network and increase mitochondrial fission [36]. Further studies are needed to investigate the mechanisms which may explore potential therapeutic strategies for dysferlinopathy.

Decreased expression of dysferlin supports the diagnosis of dysferlinopathy, but it should be noticed that the expression of dysferlin may also decrease secondary to deficiency of other related genes, such as *CAPN3* (causative gene for LGMD R1), so genetic analysis remains the definitive diagnostic criterion for dysferlinopathy. A wide range of *DYSF* variants has been identified, including missense, nonsense, frameshift deletions/insertions, splice variants and large exonic deletions [9]. Missense variants accounted for nearly half of the study in this cohort, and a comparison of the variant spectrum with a large French cohort [37] suggested a possible difference, with exonicframeshifting (18% vs. 30%) and splice (10% vs. 16%) being lower in our cohort, while missense (42% vs. 34%) and nonsense (25% vs. 20%) were more common in our cohort which also had 2 exon duplication variants. The top three outcomes in the world patients dataset were missense (42.3%), splicing (13.7%), and frameshift (11.1%) [19]. Chinese patients showed a similar pattern of variant sequence distribution as patients worldwide[17–25, 39] (Table 2). Most reported pathogenic variants for dysferlinopathy are single nucleotide variants and small insert/deletions [37], but large exonic deletions and duplications have also been described [38]. Pathogenic variants identified in this study consist of 4 (4/27) canonical-splice, 10 (10/27) nonsense, 6 (6/27) missense, and 7 (7/27) frameshift variants. Most of the variant types are single nucleotide variants consistent with previous reports. Two variants were identified previously in Chinese patients: c. 937 + 1G > A6, splicing, and c.3112 C > T (p. Arg1038Ter) [20] were also retrieved in our study. The c.3112 C > T (p. Arg1038Ter) was identified in more than one patient, and the patients who had the same variant came from different provinces, suggesting the variant may be recurrent in China. The c.2997 G > T (p. Trp999Cys) variant was the most common variant in the LGMD group in the previous study [39], but no c.2997 G > T (p. Trp999Cys) variant was observed in our cohort. Previous studies involving other genotypes have shown no observed relationship between reduced expression levels and the severity of clinical symptoms. Therefore, the effect of genotype on protein levels, and thus on phenotype, should be further investigated for each variant [39]. In addition, we also identified 15 novel variants, expanding the molecular spectrum of dysferlinopathy, and highlighted the high proportion of novel variants in Chinese patients with dysferlinopathy.

**Table 2:** Major variant types of China and the world

	missense	splice	exonicframeshifting
China	39.3%	15.3%	25.2%
World	42.3%	13.7%	11.1%

In summary, we reviewed the clinal and molecular characteristics of 26 Chinese patients with dysferlinopathy. This study showed clinical and genetic heterogeneity of dysferlinopathy and a high proportion of novel variants in the Chinese population. We also found a high rate of misdiagnosis of dysferlinopathy in primary hospitals, suggesting more attention should be paid to improving the knowledge and awareness of this disease.

## Electronic supplementary material

Below is the link to the electronic supplementary material.

Supplementary Material 1

## Data Availability

The datasets generated and/or analyzed during the current study are not publicly available due to concerns regarding patient anonymity but are available from the corresponding author upon reasonable request.

## Abbreviations

LGMD R2: Limb-girdle Muscular Dystrophy Type R2
MM: Miyoshi Myopathy
DACM: Distal anterior myopathy
PD: Proximal-Distal phenotype
PM: Polymyositis
IHC: Immunohistochemistry
HE: Hematoxylin-Eosin
MGT: Modified Gomori Trichrome
SDH: Succinate Dehydrogenase
ACP: Acid Phosphatase
NADH-TR: NADH-Tetrazolium Reductase
ORO: Oil Red O
PAS: Periodic Acid-Schiff
RRF: Ragged Red Fibers
RBF: Ragged Blue Fibers
CK: Creatine Kinase
