# Supplementary material for: The clinical, myopathological, and molecular characteristics of 26 Chinese patients with dysferlinopathy: a high proportion of misdiagnosis and novel variants
Source: BMC Neurol. 2022 Nov 1;22:398. doi: 10.1186/s12883-022-02905-w (PMC9623978; doi:10.1186/s12883-022-02905-w)
Supplement: Supplementary file 1 — Supplementary Material 1 [file 12883_2022_2905_MOESM1_ESM.docx]

**A Supplementary Table: The information and the ACMG/AMP codes**

| Patient no. | Genomic position | Nucleotide changes | ACMG/AMP codes |
| --- | --- | --- | --- |
| 1 | chr2:71708069 | c.144+1G>A | PVS1+PM2+PP4_ moderate |
|  | chr2:71753481 | c.1273+5G>C | PVS1+PM2+ PP4_ moderate |
| 2 | chr2:71747946*1 | c.965T>C | PS1+PM2+PM3+PP4_ moderate |
| 3 | chr2:71797459 | c.3026A>G | PM2+ PP4 |
|  | chr:71797809 | c.3112C>T | PVS1+PS1+PM2+PP4_moderate |
| 4 | chr2:71797809 | c.3112C>T | PVS1+PS1+PM2+PP4_ moderate |
|  | chr2:71838459 | c.3988C>T | PVS1+PS1 +PM2+PP4_ moderate |
| 5 | chr2:71801439 | c.3286C>A | PM2+PP4 |
|  | chr2:71906245 | c.5826C>A | PVS1+PM2+PM3+PP4_ moderate |
| 6 | chr2:71747339 | c.937+1G>A | PVS1+PS1+PM2+PP4_ moderate |
|  | chr2:71797407 | c.2974T>C | PS1+PM2+ PP4_ moderate |
| 7 | chr2:71753461 | c.1165G>A | PM2+PM3_Supporting+PP4 |
| 8 | chr2:71886125 | c.4756C>T | PVS1+PS1+PM2+PM3+ PP4_ supporting |
|  | chr2:7189462071894620； | c.5316dupC | PVS1+PM2+PM3+PP4_ moderate |
| 9 | chr2:71740998 | c.610C>T | PVS1+PS1+PM2+PP4_ moderate |
|  | 23-30 Exon | exon22-29  suspected duplication | PM2+PP4 |
| 10 | chr2:71791250 | c.2418C>A | PVS1+PM2+PM3_strong+PP4_ moderate |
|  | chr2:71896337 | c.5525G>A | PS1+PM2+PM3_strong+ PP4_ moderate |
| 11 | chr2:71894601 | c.5296G>A | PS1+PM2+PM3_supporting+PP4_supporting |
| 12 | chr2:71747339 | c.937+1G>A | PVS1+PM2+ PP4_ moderate |
|  | chr2:71896321 | c.5509G>A | PS1+PM2+ PP4_ moderate |
| 13 | chr2:71827854 | c.3725G>A | PS1+PP4_ supporting |
|  | chr2:71886111 | c.4742G>A | PS1+PM2+ PP4_ supporting |
| 14 | chr2:71892431 | c5197A>G | PS1+PM2+ PP4_ moderate |
|  | chr2:71894563 | c.5258A>G | PM2+PP4 |
| 15 | Chr2:71742762 | c.673C>T | PVS1+PM2+PP4_ moderate |
|  | Chr2:71906214 | c.5795T>A | PM2+PP4 |
| 16 | chr2:71709020 | c.156G>A | PVS1+PS2+PM3+PP4_ moderate |
|  | chr2:71896779 | c.5570A>G | PS1+PM2+PM3+PP4_moderate |
| 17 | chr2:71762413 | c.1375dupA | PVS1+PM2+ PP4_ moderate |
|  | chr2:71825821 | c.3648delA | PVS1+PM2+PP4_ moderate |
| 18 | chr2:71742844 | c.755C>T | PS1+PM2+ PP4_ moderate |
|  |  | Exon41-52suspected duplication | PM4+PP4 |
| 19 | chr2:71743324-71743328 | c.808_811del | PVS1+PM2+ PP4_ moderate |
|  | chr2:71797809 | c.3112C>T | PVS1+PS1+PM2+PP4_ moderate |
| 20 | chr2:71801368-71801370 | c.3216_3217delCT | PVS1+PM2+ PP4_ moderate |
|  | chr2:71891543 | c.5032T>C | PM2+PP4 |
| 21 | chr2:71891489-71891509 | c.4979_4998delGTGAGACGGTCGTCGACCTGinsA | PVS1+PM2+PM3_supporting+PP4_moderate |
| 22 | chr2:71795213 | c.2643+1G>A | PVS1+PS1+PM2+PP4_ moderate |
|  | chr2:71816726 | c.3352G>A | PM2+PP4 |
| 23 | chr2:71797407 | c.2974T>C | PS1+PM2+ PP4_ supporting |
|  | chr2:71896814-71896814 | c.5606dupG | PVS1+PS1+PM2+PP4_ supporting |
| 24 | chr2:71740998 | c.610C>T | PVS1+PS1+PM2+PM3+PP4_ moderate |
|  | chr2:71839831 | c.4228C>T | PVS1+PM2+PM3+PP4_ moderate |
| 25 | chr2:71797809 | c.3112C>T | PVS1+PM3+PM2+PP4_ moderate |
|  | chr2:71766369 | c.1480G>T | PVS1+PM3+PP4_moderate +BP4 |
| 26 | chr2:71747339 | c.937+1G>A | PVS1+PS1+PM2+PP4_ moderate |

PVS1: nonfunctional variants occur in critical genes, including nonsense, frameshift, splice, deletion/repeat and start codon variants; PS1: A variant with the same amino acid change but a different nucleotide change as a known pathogenic variant; PS3: In vivo and in vitro functional assays have established that variants cause impaired gene function. PM2: Rare or missing variants in the population database; Referring to the SVI Recommendation for in trans Criterion PM3 (Version 1.0), the PM3 score was given; The standards for PP3 recommended by the 2019 Association for Clinical Genomic Science ACGS are: REVEL≥0.7, or >2/3 of tools predicted to be harmful; For BP4, the criteria are: REVEL≤0.4, or >2/3 of the tools are predicted to be harmless and the variant position is not conservative, or no tool is predicted to be harmless; PP4: The disease associated with the variant was highly consistent with the patient's symptoms and family history.
